# Successful twin pregnancy with intracytoplasmic sperm injection using surgical sperm retrieval after 25 years of vasectomy: a case report

**DOI:** 10.5935/1518-0557.20190068

**Published:** 2020

**Authors:** Edson Borges Jr., Amanda Souza Setti, Daniela Paes de Almeida Ferreira Braga, Assumpto Iaconelli Jr.

**Affiliations:** 1 Fertility Medical Group, São Paulo, SP - Brazil.; 2 Instituto Sapientiae - Centro de Estudos e Pesquisa em Reprodução Humana Assistida, São Paulo, SP - Brazil.

**Keywords:** vasectomy, pregnancy, ICSI, PESA, azoospermia

## Abstract

The couple from this clinical case consisted of a 55 years old male with an obstructive interval of 25 years post vasectomy, and a 38 years old female partner. Both partners had normal results in infertility workup. Five mature oocytes were injected with motile spermatozoa showing morphological alterations, obtained by percutaneous epididymal sperm aspiration. Four oocytes fertilized, and three embryos were transferred with assisted hatching on day three of development, of which one was a high-quality embryo. A clinical pregnancy was confirmed by the detection of two gestational sacs with foetal heartbeats. Pregnancy was ongoing during the submission of this manuscript. The use of ICSI with PESA/TESA should be considered as a feasible alternative when vasectomy reversal fails in vasectomized men wishing to father again. Case reports like this may inspire the counseling of couples that have suffered from a previous vasectomy reversal failure and support the recommendation of ICSI with PESA treatment, which could allow those couples to have their own children, even in the presence of advanced parental age.

## INTRODUCTION

Despite the worldwide high prevalence of vasectomy, a significant fraction of the couples frequently seek medical assistance for vasectomy reversal, due to various changes in life circumstances such as remarriage, desire for fathering again or others (Patel & Smith, 2016). Intracytoplasmic sperm injection (ICSI) with surgical sperm retrieval (SSR), namely, percutaneous epididymal sperm aspiration (PESA) and testicular sperm aspiration (Abdelmassih *et al*., 2002), is one of the options for the treating those couples.

The influence of the obstructive interval (time elapsed since vasectomy) on the outcomes of ICSI with SSR has been addressed in some studies with conflicting results (Sukcharoen *et al*., 2000; Abdelmassih *et al*., 2002; Borges Júnior *et al*.,2003). This case report will describe a successful twin pregnancy obtained after 25 years of obstructive interval.

## CASE DESCRIPTION

A couple with secondary azoospermia due to vasectomy presented for surgical sperm retrieval and ICSI, in June 2018, in a private university-affiliated in vitro fertilization centre. The couple signed a written informed consent form, in which they agreed to share their treatment outcomes for research purposes. This case report was exempted from Institutional Review Board approval.

The male partner, 55 years old, father of two children from a previous marriage, had a vasectomy at the age of 29 years, resulting in an obstructive interval of 25 years. In 2017 a vasectomy reversal was attempted, however, two azoospermic samples were obtained after surgery. Hormonal profile, karyotype, physical examination (testis volume and consistency, presence and consistency of the vasa deferentia, consistency of the epididymis, and the presence of varicoceles), and testicular ultrasound evaluation results were normal. Female partner was 38 years old. Hormonal profile, karyotype, physical examination, hysterosalpingography, hysteroscopy and transvaginal ultrasonography results were normal.

Ovarian stimulation was achieved with 300 IU daily recombinant follicle-stimulating hormone (r-FSH, Gonal-F(r), Serono, Geneva, Switzerland) subcutaneous injections. Pituitary suppression was achieved with 0.25 mg daily gonadotropin-releasing hormone (GnRH) antagonist, ganirelix acetate (Orgalutran; NV Organon, Oss, The Netherlands). Ovulation was triggered with recombinant human chorionic gonadotrophin (hCG, Ovidrel(tm), Serono, Geneva, Switzerland) when at least three follicles ≥ 17 mm were observed. Oocyte retrieval was performed 35 h later. A total of 8 oocytes were retrieved, of which 5 were in metaphase II (MII) stage, and 3 in prophase I stage.

The PESA was performed on the same day by a consultant urological surgeon and a senior clinical embryologist, under local anesthetic block. Briefly, a 27.5-gauge needle was introduced into the head of the epididymis, followed by a delicate aspiration using a 1 ml syringe containing buffered supplemented culture medium (Global w/HEPES, LifeGlobal). The aspirate was transferred to a dish and examined for spermatozoa immediately by the embryologist. Four punctures were performed in the left epididymis, with no sperm yield. Then, four punctures were performed in the right epididymis, yielding immotile sperm and six motile spermatozoa. Then, percutaneous testicular sperm aspiration was performed, using a 19-gauge needle, using syringe with positive pressure. Four testicular fragments were aspirated from different directions through the right testis, yielding immotile sperm only. The sample from the right testis PESA was prepared using simple washing, and stored at 37 ºC until the ICSI procedure.

Five hours after oocyte retrieval, five MII oocytes were injected, according to the technique described by Palermo *et al*. (1992) with motile spermatozoa showing morphological alterations.

The normal fertilization of four oocytes (80.0%) was confirmed 16 hours post-ICSI. One high-quality and two low-quality embryos were transferred after assisted hatching. Embryos were transferred on day 3 of development using a soft catheter. The luteal phase was supported by intravaginal progesterone 400 mg (Utrogestan) twice a day and transdermal estradiol 200 mcg (Estradot), one adhesive every three days. A pregnancy test was performed 12 days after embryo transfer yielding a positive result. A transvaginal ultrasound scan was performed 2 weeks later, and a clinical pregnancy was diagnosed upon the detection of two gestational sacs with foetal heartbeats. Spontaneous miscarriage was discarded after 20 weeks of pregnancy. Pregnancy was ongoing during the submission of this manuscript.

## DISCUSSION

The use of PESA/TESA combined with ICSI is an effective treatment option for obstructive azoospermia. We reported a case in which a twin a pregnancy was achieved with PESA/TESA and ICSI in a couple after 25 years of obstructive interval. Previous studies have demonstrated that the obstructive interval negatively influences pregnancy (Abdelmassih *et al.*, 2002; Borges Júnior *et al*., 2003), ongoing pregnancy (Abdelmassih*et al*., 2002; Borges Júnior *et al*.,2003), implantation (Abdelmassih *et al*., 2002; Borges Júnior *et al*., 2003) and miscarriage (Borges Júnior *et al*.,2003) rates. One study, with severe limited power, failed to demonstrate such associations (Sukcharoen *et al*., 2000). A recent study from our group showed that the obstructive interval negatively influenced not only the SSR outcomes but also the blastocyst development, implantation and pregnancy rates. In addition, a negative predictive value on the chance of pregnancy was observed with > 17 years of vasectomy, despite pregnancies had been achieved with longer obstructive intervals (manuscript in press).

Vasectomy reversal is another option for vasectomized patients who want to father again, but it may not restore male fertility due to several repercussions of vasectomy such as (i) secondary epididymal blockage and anti-sperm antibody formation (Sukcharoen *et al.*, 2000), (ii) endocrine disruption leading to Sertoli cell vacuolation and dysfunction (Kubota, 1969), (iii) oxidative stress in sperm cells (Shapiro *et al*., 1998; Kolettis *et al*., 1999; Practice Committee of the American Society for Reproductive Medicine, 2015) increased interstitial fibrosis in testes (Shiraishi *et al*., 2003), and (v) increased apoptosis in the seminiferous tubules, Sertoli cells, primary spermatocytes, and round spermatids (O'Neill *et al.*, 2007). The use of PESA or TESA and ICSI is supposed to bypass those factors that interfere with fertility in vasectomized men.

Though, the success of any fertility treatment relies on both partners, thus, factors like maternal age and associated female infertility must be taken into account. The female partner here presented was 38 years, which is considered an advanced maternal age, and had a poor ovarian response to stimulation (5 MII oocytes retrieved), despite no sign of infertility or low antral follicle count. In addition, only one transferred embryo was of high quality. Surprisingly, a successful twin pregnancy was achieved and in ongoing without complications.

The use of ICSI with PESA/TESA should be considered as a feasible alternative when vasectomy reversal fails in vasectomized men wishing to father again. Case reports like this may inspire the counseling of couples that have suffered from a previous vasectomy reversal failure and support the recommendation of ICSI with PESA treatment, which could allow those couples to have their own children, even in the presence of advanced parental age.
